# In-depth immunochemical characterization of the serum antibody response using a dual-titration microspot assay

**DOI:** 10.3389/fimmu.2025.1494624

**Published:** 2025-02-25

**Authors:** Ágnes Kovács, Zoltán Hérincs, Krisztián Papp, Jakub Zbigniew Kaczmarek, Daniel Nyberg Larsen, Pernille Stage, László Bereczki, Eszter Ujhelyi, Tamás Pfeil, József Prechl

**Affiliations:** ^1^ Department of Biostatistics, University of Veterinary Medicine Budapest, Budapest, Hungary; ^2^ Department of Applied Analysis and Computational Mathematics, Eötvös Loránd University, Budapest, Hungary; ^3^ R&D Laboratory, Diagnosticum Zrt, Budapest, Hungary; ^4^ Research and Development Department, Ovodan Biotech A/S, Odense, Denmark; ^5^ Department of Biochemistry and Molecular Biology, University of Southern Denmark, Odense, Denmark; ^6^ G1 Labordiagnosztikai Kft, Budapest, Hungary; ^7^ HUN-REN–ELTE Numerical Analysis and Large Networks Research Group, Budapest, Hungary

**Keywords:** antibody, serology, microarray, chemical thermodynamics, curve fitting, SARS-CoV-2, quantitative systems biology

## Abstract

Antigen specific humoral immunity can be characterized by the analysis of serum antibodies. While serological assays for the measurement of specific antibody levels are available, these are not quantitative in the biochemical sense. Yet, understanding humoral immune responses quantitatively on the systemic level would need a universal, complete, quantitative, comparable measurement method of antigen specific serum antibodies of selected immunoglobulin classes. Here we describe a fluorescent, dual-titration immunoassay, which provides the biochemical parameters that are both necessary and sufficient to quantitatively characterize the humoral immune response. For validation of theory, we used recombinant receptor binding domain of SARS-CoV-2 as antigen on microspot arrays and varied the concentration of both the antigen and the serum antibodies from infected persons to obtain a measurement matrix of binding data. Both titration curves were simultaneously fitted using an algorithm based on the generalized logistic function and adapted for analyzing biochemical variables of binding. We obtained equilibrium affinity constants and concentrations for distinct antibody classes. These variables reflect the quality and the effective quantity of serum antibodies, respectively. The proposed fluorescent dual-titration microspot immunoassay can generate truly quantitative serological data that is suitable for immunological, medical and systems biological analysis.

## 1 Introduction

The adaptive immune system maintains host integrity by controlling the levels of molecules and cells over a very wide range. The humoral adaptive immune system achieves this by employing effector molecules with tunable specificity and efficiency. These molecules, antibodies (Ab), evolve with the help of B lymphocytes that use them both as sensors for their survival and as effectors for the removal of target molecules. To protect against viral invasion of host cells, antibodies with enhanced effector activity (1) are produced and persist over time (2). However, the emergence of novel viruses with enhanced ability to enter and spread in the host may overwhelm before such responses might take place (3). Since antibodies circulating in blood are good indicators of the status of humoral immunity, the characterization of serum antibodies, Ab serology, plays a critical role in immunodiagnostics.

Currently two main approaches dominate serological methods of antigen (Ag) specific Ab measurements: titration and single-point assays. Titration is the use of a series of gradually diluted serum and the determination of mid-point or end-point titer from the dilution curve. This approach does incorporate the measurement of effects due to changing reaction conditions, but the readout most often neglects curve shape and focuses on a single unitless parameter: the titer. Single-point measurements are optimized for diagnostic power (sensitivity and specificity) (4–6) and neglect potential effects of serum dilution. The readout of single-point measurements is an arbitrary though standardized unit of reactivity. Virus neutralization (7) and surrogate neutralization assays (8, 9) can also be carried out in the single-point or titration format. What is common in these assays is that none of them varies or takes into account the density of the target molecule used in the assay: the solid-phase coupled Ag or the virus receptor on cells.

Interactions between Ab and Ag constitute the basis of immunoassays and while several physical and mathematical models have been developed and utilized to characterize them, yet these are primarily assays wherein the analyte is the Ag and determination of Ag concentration is the aim. Less attention has been paid to the development of models dedicated to the special requirements of circulating serum Ab detection, that is, when the analyte is not a single molecular species but the collection of serum Ab. Serum Abs are highly heterogeneous with respect to epitope recognition, affinity, concentrations and structure. A blood serum sample in fact reflects immunological memory of past infections, diseases, along with current immunological activity. While serological assay results are given in units of activity, referring to binding or biochemical activity, no clear relationship to thermodynamic activity has been established (10). Early approaches of Ag-Ab interaction modeling, based on predictions of molecular properties (11), suggested that too many physical properties of the interacting molecules should be estimated.

The difficulty of serum Ab quantification lies in the fact that we are dealing with two unknown variables: average effective affinities and concentrations of Abs that bind to the Ag in the assay. Any approach aiming to quantify serum Abs should consider estimating both. Traditionally equilibrium dialysis was the method of choice for measuring affinity constants in solution when radioimmunoassays were used. These assays used a constant concentration of Ab (e.g., 1:100 dilution of serum) and varying concentrations of Ag, calculating the concentration of free radioactively labeled Ag, once equilibrium was established, from the radioactivity. The Scatchard plot was used for calculating the median and mean equilibrium dissociation constant (KD) (12) and for the assessment of affinity heterogeneity and the Sips plot was used for quantifying heterogeneity (13, 14). Current approaches employ novel technologies for assessing affinity and concentration. Lippok et al. used microscale thermopheresis to measure both affinity and concentration of polyclonal Abs in solution (15). Fiedler et al. utilized diffusional sizing of labeled Ag in a microfluidic device for KD and concentration estimation (16). Tang et al. described the combination of ELISA and quantitative mass spectrometry, an approach that allows quantitation of bound Ab (17). These novel approaches generate results in universal biochemical units but still fall short of revealing important biochemical properties of serum Abs of selected classes.

Microspot immunoassays represent special measurement conditions with respect to the relative amounts of the reactants: the mass of the reactant in the microspot is negligible to the mass of the reactant in solution. This property is exploited in ambient analyte immunoassays (18), where capture Abs are printed as microspots and are used for determining the concentration of Ag in solution. Because of the negligible concentration change caused by the capture of Ag from solution, this setup is ideal for concentration measurements (19). Microspotted Ag can be used for the measurement of serum Ab binding, with the assumption that captured Ab causes negligible change in the composition of the solution (20). From the physico-chemical point of view, these conditions correspond to measurements carried out at infinite Ag dilution.

As a model system for our proof-of-concept study we chose to measure the serum Ab response against a domain of the spike protein of the pandemic coronavirus. Infection with SARS-CoV-2 results in the appearance of IgM (21), IgG (22–24) and IgA (25) directed against various viral components (26–29). The response builds on immunological imprints from common cold viruses (30), is connected to disease severity (31–35) and lasts several months (36, 37). Vaccination also induces Ab production, therefore serological measurements can help assess vaccine immunogenicity and estimate protectivity (38–41), durability (42) and cross-reactivity with emerging new variants (43). The various indicators of humoral immune response, such as titers (44, 45), avidity (46), glycosylation (33) of virus specific Abs are all related to clinical aspects of the infection and disease, but these relationships are highly variable, not definite (3), partially because of the difficulties in comparing different measurement methods (32).

We recently developed a microspot-based, dual-titration immunoassay for the estimation of affinity distribution for multiple immunoglobulin classes (20) utilizing an advanced fitting algorithm (47). Here we describe a further improvement of the assay and demonstrate its applicability for the characterization of anti-SARS-CoV-2 Ab response, by the estimation of chemical thermodynamic variables of SARS-CoV-2 RBD specific Abs of various classes. This approach not only produces universal biochemical units of measurement for the Ab isotype of interest but at the same time also provides insight into the chemical thermodynamics of a complex system.

## 2 Materials and methods

### 2.1 Serum samples

Commercially available serum from confirmed COVID-19 positive and negative subjects with available IgG and IgM test results (RayBiotech CoV-PosSet-2) was obtained from THP Medical Products Vertriebs GmbH, Vienna, Austria. COVID-19 positive samples were tested by the manufacturer using their COVID-19 ELISA kits for IgG and IgM measurements (RayBiotech, Georgia, USA).

### 2.2 Antigen production

#### 2.2.1 Gene synthesis, protein expression and characterization of SARS-CoV-2 Spike RBD 319-541

SARS-CoV-2 Spike RBD 319-541 sequence was based on the first strain of SARS-CoV-2 isolated from a clinical patient on January the 6th 2020, (GISAID: EPI_ISL_402119). The sequence was synthesized to include the Tyr-Pho signal peptide and a N-terminal hexahistidine (6xHIS) tag. The construct was synthesized and cloned commercially into pcDNA3.4-TOPO plasmid (Life Technologies B.V. Europe). SARS-CoV-2 Spike RBD 319-541 recombinant protein (OBA0101, Ovodan Biotech) was expressed in 25 mL culture using the Expi293F expression system (#A14635; ThermoFisher Scientific) according to manufacturer’s instructions. Expressed proteins were harvested by centrifugation 6 days post transfection and immediately purified from the supernatant on a Ni-NTA Superflow column (#30430, Qiagen). Eluted protein fraction was buffer exchanged into phosphate-buffered saline solution pH 7.4 using a HiPrep 26/10 Desalting column (#GE17-5087-01, Cytiva) and stored at -20°C.

The obtained protein fraction was subjected to Sodium Dodecyl Sulfate Polyacrylamide gel electrophoresis (SDS-PAGE) using RunBlue (#ab270467 Abcam) 4-12% Bis-Tris polyacrylamide gel. Prior to loading, the samples have been mixed with 2,5µl NuPAGE LDS (4x) sample buffer (Life Technologies) each and incubated in 70°C for 10 minutes in a glass container filled with water, heated on a VMS-C7 heating block (VWR). Afterwards the samples have been briefly centrifuged using MiniSpin Plus Centrifuge at 1000 RPM for 15 seconds (Eppendorf). Electrophoresis was performed by using XCell SureLock Electrophoresis Cell (Novex) and Easy Power 500 (Invitrogen) in a non-reduced environment for 45 minutes at 200V and 110mA. Electrophoresis was carried out in a 1X MOPS-SDS Buffer (VWR). Staining was performed by Coomassie Simply Blue Safe Stain (#LC6060 Invitrogen) for 1 hour on a PS-M3D orbital shaker (Grand Bio). The gel was destained in deionized water overnight and visualized by using Bio-Rad Gel Doc XR – Molecular Imager. The molecular weight marker used was peq Marker Gold V (VWR). The detailed protocol and PAGE image are available as **Supplementary Data**.

#### 2.2.2 SARS-CoV-2 recombinant RBD sequence characterization by mass spectrometry

A total of 20 µg of SARS-CoV-2 Spike RBD 319-541 was reduced with dithiothreitol (20 mM) for 30 minutes at 57 degrees and alkylated with iodoacetamide (54 mM) for 20 minutes at room temperature and in the dark, the reaction was stopped with dithiothreitol. The reduced and alkylated SARS-CoV-2 Spike RBD 319-541 was split into two batches, where the first was treated with 2% homemade methylated trypsin [1] for an hour at 57 degrees and PNGaseF (0.5 µl) (Promega, V4831) for an hour at 37 degrees, while the other was only treated with 2% trypsin for an hour at 57 degrees.

The two batches were micro-purified prior to analysis by mass spectrometry. The micro-purification was performed in accordance with Rappsilber et al. (48), where a p200 tip was plugged with M3 material [Empore octyl C8, 66882-U] and 1 μl of R2 material [Poros 20 R2 Applied Biosystems, Part no. 1-1129-06] was added. The stage tip was then activated using 100% acetonitrile [VWR, 83640.290], followed by equilibration with 0.1% TFA [MERCK, 200-929-3], sample was then 1 μg of sample was loaded into 40 μl of 0.1% TFA, followed by washing with 0,1% TFA. The sample was then eluted from the stage tip using first 50% acetonitrile, 0.1% TFA and secondly using 70% acetonitrile, 0.1% TFA. The eluted samples were then lyophilized using an Eppendorf vacuum centrifuge [VWR, 20318.297], prior to running the samples were resuspended in 6 μl 0.1% formic acid [maker needed]. Both batches were run in duplicate, using 1 μg of sample per run, with a standard liquid chromatographic tandem mass spectrometric analysis on an Orbitrap Exploris™ 480 Mass Spectrometer from Thermo Fisher Scientific. The follow main settings were used; MS1 resolution: 120000, scan range (m/z): 350-1400, included charge state(s): 1.0e4, dynamic exclusion after 1 time with an exclusion time of 30 seconds, MS2 resolution: 30000, isolation window MS2 (m/z): 0.8, first mass MS2 (m/z): 110, data type: centroid.

The data files from the mass spectrometer were converted using MSConvert from ProteoWizard [https://proteowizard.sourceforge.io/], followed by data search and analysis in GPMAW from Lighthouse Data [http://www.gpmaw.com/].

The mass spectrometry proteomics data have been deposited to the ProteomeXchange Consortium via the PRIDE (57) partner repository with the dataset identifier PXD040415. Further details of protein characterization are available online as **Supplementary Data**.

### 2.3 Dual-titration microspot immunoassay

Maps of the layout of slides and subarrays, along with a detailed description of the protocol are available online as **Supplementary Data**.

#### 2.3.1 Microarray production

Experiments were carried out on hydrogel-coated glass slides (Nexterion Slide H, Schott Minifab, Jena, Germany) by using a BioOdyssey Calligrapher MiniArrayer (BioRad, Hercules, CA, USA). A 14-point dilution series of RBD was prepared with a combination of a ½ and ⅓ diluting series, and spotted on slides in triplicates. The final concentration gradient steps were: 16.66, 8.33, 5.55, 4.16, 2.08, 1.85, 1.04, 0.61, 0.52, 0.26, 0.20, 0.13, 0.068 and 0.065 µM. Slides were dried for 1h at 37°C then soaked in 0.1 M Tris buffer (pH=8.0) for 1h at 37°C in order to block reactive residues on the surface. Once prepared, slides were kept in sealed non-transparent bags at 4°C.

#### 2.3.2 Sample handling and signal detection

Dried arrays were rehydrated in 110 µl PBS (3×5 minutes) before using, then sub-arrays were incubated in 70 µl diluted sample at 37°C for 1 hour. Sample dilution was carried out in PBS-BSA-Tween (PBS, 0.5% BSA, 0.05% Tween 20). Serum treated slides were washed in 0.05% Tween-PBS, then incubated at room temperature for 30 minutes with fluorescently labeled Abs that were diluted in the blocking buffer (0.05% Tween 20, 2% BSA, PBS). The first mix of detecting Abs was composed of the following: anti-human-IgG F(ab’)_2_ – Alexa488 (Jackson, Ref.:109-646-097), anti-human-IgA – Alexa647 (Jackson, Ref.:109-606-011), anti-human-IgM – Cy3 (Jackson, Ref.: 109-166-129). Chips were washed again and following drying, slides were scanned using SensoSpot fluorescent microarray scanner (Sensovation AG, Stockach, Germany). Fluorescence signals below ln(FI)=4 were excluded from further analysis.

#### 2.3.3 Analysis of the microarray data

Images of the slides were analyzed with GenePix Pro 6.0 software after visual inspection. Spots were recognized, aligned and analyzed by the program, then gpr files containing the spot data were created. Relative fluorescence intensity (RFI) values were calculated for each spot using the feature’s median RFI value of which the feature’s local background was subtracted individually for each feature. Further analysis was carried out by using the statistical programming environment R (version 3.5.2).

### 2.4 Curve fitting

The general theoretical approach to data analysis was similar to that described recently (20). We use a linear model for polyclonal reactions (49) taking into consideration that a bound Ab inhibits nearby free Ags from forming complexes with other Abs such that the concentration of immune complexes is a logistic function of the logarithm of the total Ag concentration (Equations 1–6). Since the logarithm of fluorescent intensity is proportional to the logarithm of bound Ab concentration, we assume that the fluorescent intensity of detected Abs is a Richards function (R) of the logarithm of total Ag concentration (see **Supplementary Text 1** of (20)), with the following parametrization:


(1) $$R\left(x\right)=A*{\left(1+\nu e^{-k\left(x-x_{i}\right)}\right)}^{-\;\frac{1}{\nu }}$$

with k = 1, where x is logarithm of Ag concentration ln[Ag], A is the signal corresponding to total Ab concentration [Ab] (limit of function R(x) at infinity), x_i_ is the inflection point of function R, ν is the asymmetry parameter.

The upper limit of the fluorescence, A, depends on the dilution of serum: the less diluted the serum sample, the higher the Ab concentration. We used the reciprocal of serum dilution as a surrogate Ab concentration. Ten times diluted serum corresponds to a concentration of 0.1. The fluorescent signal intensity of bound Ab is thus determined by both Ag and Ab concentrations, which we express using the logarithm total Ag concentration, x=ln[Ag], and the logarithm of the reciprocal of serum dilution factor, y=ln[Ab], as the product of two generalized logistic functions (R_1_(x)*R_2_(y)) with k=1 in the form


(2) $$r\left(x,y\right)=C*{\left(1+\nu_{1}e^{-\left(x-x_{i}\right)}\right)}^{-\frac{1}{\nu_{1}}}*{\left(1+\nu_{2}e^{-\left(y-y_{i}\right)}\right)}^{-\frac{1}{\nu_{2}}}$$

where C is the fluorescent signal corresponding to the maximal concentration of AbAg complexes. Both terms can be normalized to their own inflection points.


(3) $$r_{n}\left(x,y\right)=C_{n}*{\left(\frac{1+\nu_{1}}{1+\nu_{1}e^{-\left(x-x_{i}\right)}}\right)}^{\frac{1}{\nu_{1}}}*{\left(\frac{1+\nu_{2}}{1+\nu_{2}e^{-\left(y-y_{i}\right)}}\right)}^{\frac{1}{\nu_{2}}}$$

with the visual advantage of moving x_i_ and y_i_ to the same horizontal line z=1. In this form C_n_ corresponds to the fluorescent signal of AbAg complexes at the inflection point of both functions.

A logarithmic transformation converts the proportional variance pattern to a constant variance pattern and thus the conversion makes the transformed data more suitable for fitting the model. The above multiplicative relationship then changes to an additive one (lnR_1_(x)+lnR_2_(y)) in the form of


(4) $$ln\;r_{n}\left(x,y\right)=ln\left(C_{n}\right)+\frac{1}{\nu_{1}}ln\left(\frac{1+\nu_{1}}{1+\nu_{1}e^{-\left(x-x_{i}\right)}}\right)+\frac{1}{\nu_{2}}ln(\frac{1+\nu_{2}}{1+\nu_{2}e^{-\left(y-y_{i}\right)}}).$$

In order to reduce the number of estimated variables and to introduce a variable common to both titration curves, we used the relationship between the asymmetry parameters of Ab and Ag titration curves (Prechl 2024):


(5) $$v_{2}=\frac{1}{\nu_{1}}-1.$$

This normalized, generalized logistic model on the log-log scale was fit to the data in the following form:


(6) $$ln\left(FI\right)=ln\left(C_{n}\right)+ln\left\{{\left(\frac{1+\nu_{Ag}}{1+\nu_{Ag}e^{-k\left(ln\left[Ag\right]-ln\left[Ag\right]{}^{\circ}\right)}}\right)}^{\frac{1}{\nu_{Ag}}}\right\}+ln\left\{{\left(\frac{1+\nu_{Ab}}{1+\nu_{Ab}e^{-k'\left(ln\left[Ab\right]-ln\left[Ab\right]{}^{\circ}\right)}}\right)}^{\frac{1}{\nu_{Ab}}}\right\}$$

and parameter estimates and 95% confidence intervals were obtained using the R software (version 3.5.2). Nonlinear least squares estimates for the model parameters were calculated using the Gauss-Newton algorithm of the *nls* function from the statistical software package R (version 3.5.2). Confidence intervals generated for the model parameters were based on the Wald-based technique.

### 2.5 Statistical analysis of results

Throughout the paper we use ln for natural logarithm and log for base 10 logarithm. While curve fitting was carried out on ln transformed data, for the visualization and comparative analysis of results we use log data as is conventional in immunochemistry. Linear regression was used to obtain equations for the calibration of fluorescence and Ab concentrations of IgA, IgG and IgM. Random effects models were used to analyze ν, log(KD), log(KD’_Ag_), log[AbAg]°, and log([Ab]/KD), with group (positive, negative), antibody class (IgA, IgG, and IgM) and their interaction as fixed effects, and with patient ID as a random effect. Tukey-adjusted p-values were calculated for multiple comparisons between the two groups at each level of anybody class. The statistical analysis was performed using the glht() function from the R multcomp package (R version 4.3.2).

## 3 Results

### 3.1 Definition of standard reference state of polyclonal Ab interactions

In chemical thermodynamics the standard state is a precisely defined reference state, which serves as a common reference point for the comparison of thermodynamic properties. The standard chemical potential is arbitrarily defined in any system in a way to suit the description of the system. For the purpose of chemical reactions pressure, temperature and material composition can define a standard state. For the purpose of an immunoassay composition is critical: the chemical potential of an Ab solution, besides the affinity and concentration of the Ab itself, is determined by the quality and the concentration of the target molecules, Ag. In our model Ab binding to Ag microspots is described by the product of two Richards functions (20). Each of the Richards functions represents the growth of the relative concentration of bound reactants, one as a function of the logarithm of Ag density, the other as a function of the logarithm of Ab concentration. In this system the inflection points of the two functions can serve as the origin of coordinates. We therefore define the standard reference state of a particular serum Ab and particular Ag mixture as where the concentrations of free Ab and Ag are equal to the KD and the concentration of AbAg complex in the standard state is [AbAg]°. In general, it means that the interaction of each serum with a given Ag will have a distinct deviation from the standard state and the identified asymmetry parameters will therefore characterize the serum sample in terms of its thermodynamic activity against the tested Ag. We shall use the degree sign (°) to indicate standard reference state conditions.

**Figure 1:**
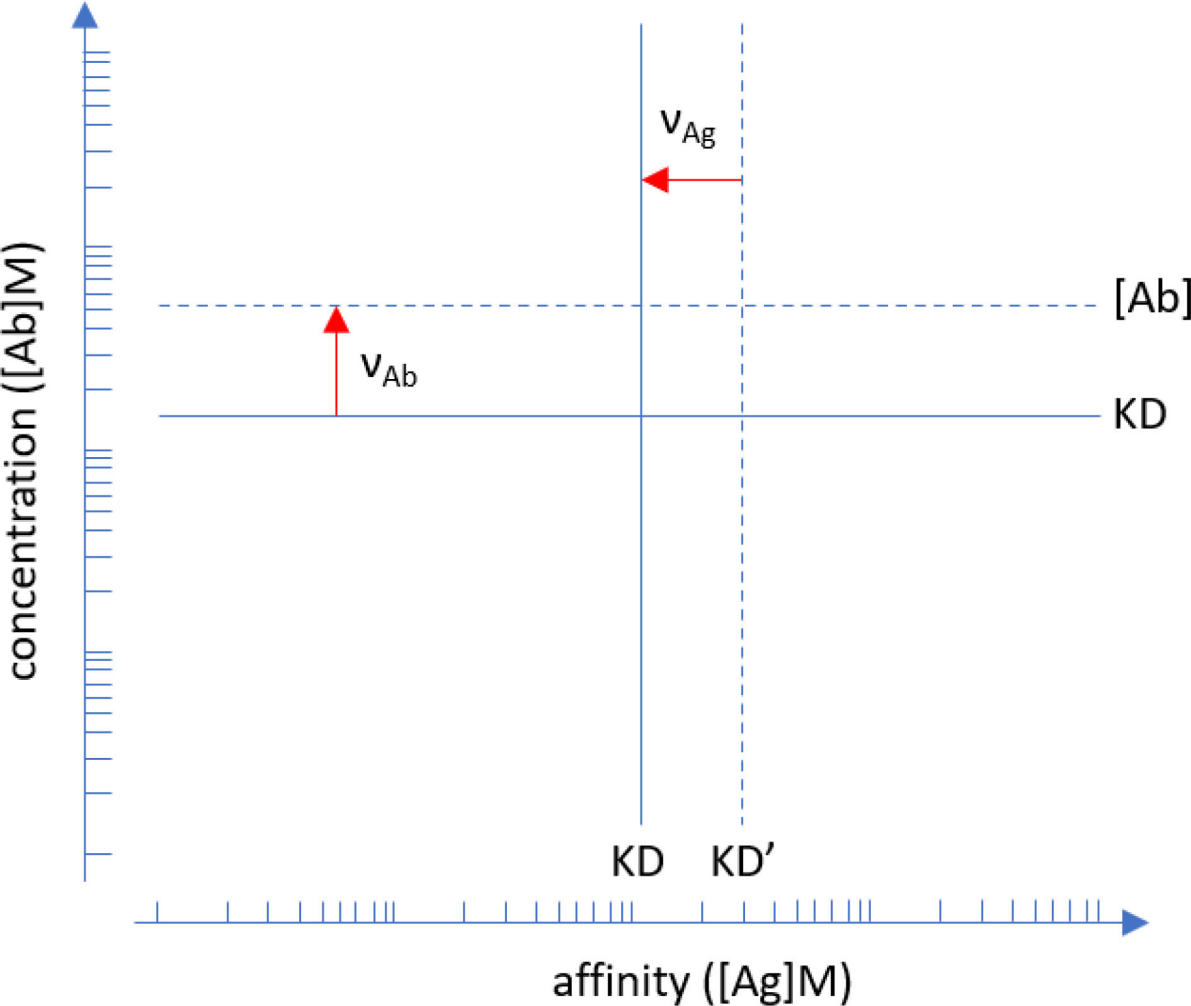
Interpretation of asymmetry parameters $\nu_{Ag}\;and\;\nu_{Ab}$ . Points of inflection identified by the fitted Richards functions are shifted away from ideality, which is represented by the logistic function. Apparent affinity is lower (KD’>KD) than true affinity (solid line), the red arrow indicates the shift. Serum Ab concentration can be lower or higher than the KD, only the latter is indicated by the red arrow for clarity. Units are in moles/liter. Please note that the plot is in log-log scale so asymmetry parameters are multiplication factors.

The logistic function defines an ideal reaction where the decrease of free binding partner is symmetric to the increase of the bound form. Microspot immunoassay conditions are non-ideal but rather limiting conditions: Abs bind to the microspot with negligible change in their composition while reaching equilibrium. The Richards function, a form of generalized logistic function, describes non-ideality by allowing asymmetry in these curves and the asymmetry is captured by this additional ν parameter. In physical chemistry the variable that adjusts concentration to thermodynamic activity by accounting for non-ideality in the reaction is the thermodynamic activity coefficient. This activity coefficient therefore changes as the concentration and interactions of Abs change during titration. The observed asymmetric titration curve characterizes the extent of this deviation from ideality (**Figure 1**).

Overall, in our model the equilibrium concentration of [AbAg] for each [Ab] and [Ag] composition is obtained by the calculation of the standard state concentrations [AbAg]°, [Ab]°, [Ag]° and the asymmetry parameters from the estimated values of fitting variables.

### 3.2 Calibration of measurement

**Figure 2:**
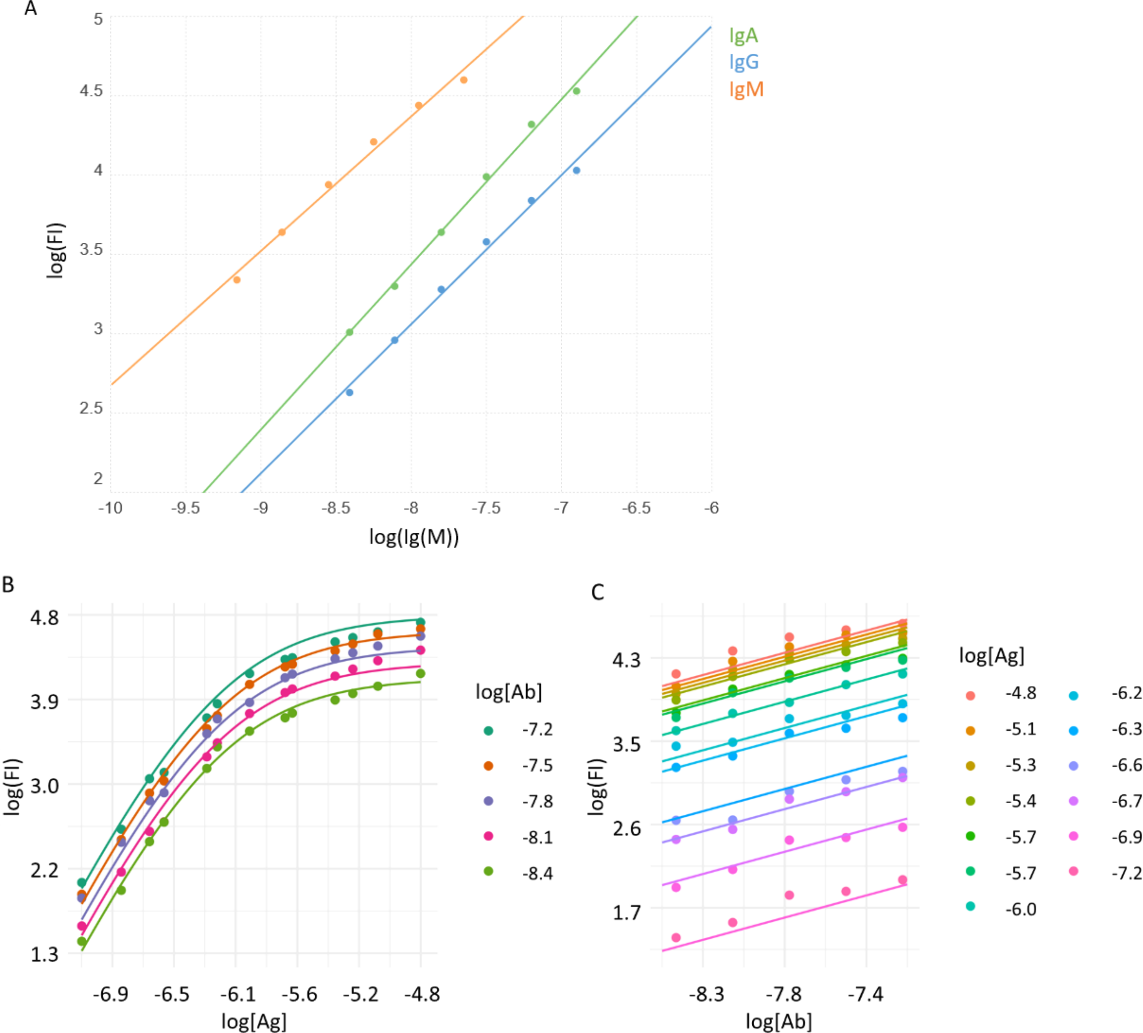
Calibration of Ab signals and curve fitting. **(A)** A mixture of purified immunoglobulins was used to relate fluorescent signals of binding (FI) to molar concentration, using linear regression. Averaged signals of printed Ig mix from all subarrays are represented by dots, lines stand for the fitted function. B) A monoclonal Ab specific for the hexahistidine tag in the recombinant viral RBD was used to characterize the measurement system. Measurement data (dots) and fitted curves for Ag **(B)** and Ab **(C)** titration curves are shown.

Each subarray of the microarray slide contained a dilution series of a mixture of purified human IgM, IgG and IgA, which we used as reference measurement points for calibration. Calibration curves obtained by fitting linear regression curves to log-log datasets (**Figure 2A**) showed strong and closely linear correlations in this measurement range for IgA (slope=1.05, R^2^ = 0,99), IgG (slope=0.95, R^2^ = 0.99) and IgM (slope=0.85, R^2^ = 0.99). These curves establish the relationship between FI and Ab concentrations and were used for calculating [AbAg]°. Equations for the calculation are available in the **Supplementary Data**.

We used the monoclonal Ab AD1.1.10, specific for the hexahistidine tag engineered to the C-terminus of the recombinant RBD, to examine the performance of the measurement system and the fitting strategy with an Ab of known concentration. A series of different concentrations of the Ab was reacted with the microarray and detected using an anti-mouse IgG secondary reagent. The binding data was then fitted using the above algorithm (**Figures 2B, C**). The calibration results indicated that the measurement system is suitable to examine Ab reactivity in the low nanomolar to micromolar [Ab] range, using microspotted [Ag] in a similar nanomolar to micromolar range.

### 3.3 Complex formation in microspots and in ELISA

When serum Abs are reacted with Ag, the amount of generated AbAg complexes depends on the reaction conditions, primarily the applied concentration of the reactants. This is one of the reasons why different Ab serological tests cannot be compared. By defining the standard reaction conditions as those with standard concentration of both Ab and Ag ([Ab]=KD, [Ag]=KD) in a measurement system where Ab is in huge excess, we can calculate the standard concentration of complexes: [AbAg]°. Its value can be calculated from the calibration curves and ln(C_n_), and represents a reference point from which [AbAg] values deviate depending on ln[Ab] and ln[Ag].

**Figure 3:**
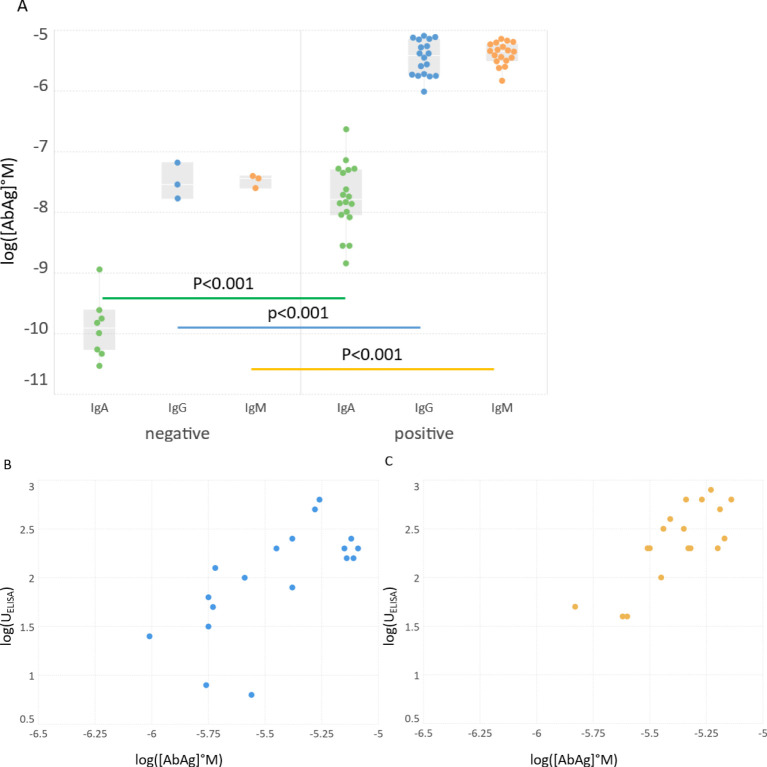
Standard complex concentrations in the groups and their relationship with ELISA results. **(A)** Comparison of standard concentrations in between groups and Ab classes. **(B, C)** Logarithmic values of ELISA results of positive sera were correlated with the obtained log[AbAg]°concentrations. ELISA results for IgA were not available. Significance of differences is characterized by the shown p value above the bars connecting box plots.

We measured the binding of IgA, IgG and IgM to recombinant SARS-CoV-2 RBD in serum samples from COVID-19 negative and positive individuals. Only samples with sufficient datapoints could be fitted by the algorithm, from the 10 negative samples only those with strong enough binding signals could be fitted (negative group: IgA, n=8; IgG, n=3; IgM, n=3). Two samples from the seropositive set were excluded from the analysis because the delay from COVID-19 onset to blood sampling was too short (<14 days) and accordingly the obtained measurement data were outliers in the group, so the analyzed data contained 18 samples for all three Ab classes which were all fitted successfully (positive group: n=18). There was a significant increase of [AbAg]° values in the positive group that was similar in all three measured Ab classes, reflecting an immune response with increased IgA, IgG and IgM binding to the viral protein in the infected individuals (**Figure 3A**). We compared the available ELISA results of the positive group to our microarray-derived [AbAg]° data. The logarithm of ELISA units were positively correlated with the logarithm of standard complex concentrations for IgG (R^2^ = 0.48, p=0.0014) and IgM (R^2^ = 0.61, p=0.0001) (**Figures 3B, C**).

### 3.4 Calculation of the affinity and relative concentration of serum Ab

The application of Richards function for data fitting allows for deviation from ideality, the extent of deviation is measured by the asymmetry parameters, which then allow to characterize non-ideality by biochemical variables. The real KD is calculated from the observed apparent KD’_Ag_,: at a distance of $\text{ln}\left(\nu_{Ag}\right)$ from the inflection point of Ag titration is the ideal concentration ln[Ag]° at which half of the binder Ab would be saturated and therefore [Ag]°=KD. Under our asymmetric microspot measurement conditions, the point of inflection shifts depending on the effective serum Ab concentration relative to the KD. Thus, when [Ab]=KD then half of Ag is saturated and the inflection point is exactly at the undiluted serum measurement point. When [Ab]>KD the inflection point shifts to the left by $\text{ln}\left(\nu_{Ab}\right)$ and falls on the titration curve; when [Ab]<KD we observe no inflection because it falls beyond the titration curve. Thus, asymmetry parameter $\nu_{Ab}$ is the ratio [Ab]/KD, a relative concentration index, which we call the thermodynamic titer.

**Figure 4:**
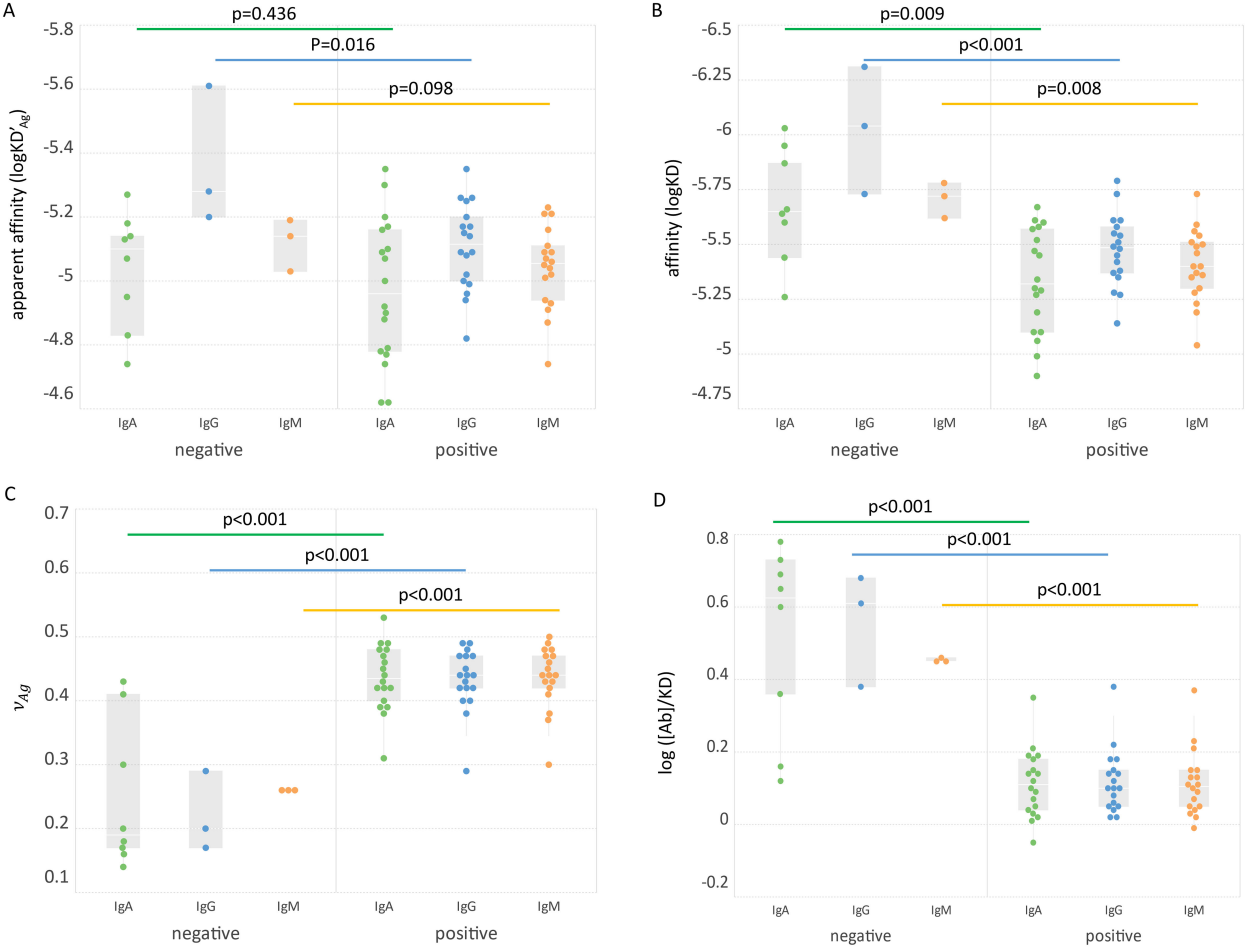
Comparison of groups based on biochemical variables. The apparent affinity **(A)**, affinity **(B)**, asymmetry parameter **(C)** and relative concentration **(D)** of three Ab classes in the two groups are shown. Significance of Ab class group differences (bars connecting box plots) is characterized by the shown p values.

We can now define besides the qualitative parameters of apparent and true KD the quantitative parameter that is universally comparable and can characterize serum Ab reactivity. The calculated physico-chemical variables of RBD specific IgA, IgG and IgM in the two groups were compared (**Figure 4**). While KD’_Ag_ differences did not reach significance, KD, $\nu_{Ag}$ and [Ab]/KD were significantly different for all the Ab classes between the two sample groups.

## 4 Discussion

Conventional approaches to the qualitative and quantitative characterization of specific serum Abs yield arbitrary units or titers. To obtain biochemically meaningful, truly quantitative results, we applied physical chemistry in the interpretation of microspot Ag titration results. First, we defined the standard chemical thermodynamic state of a particular Ag molecule in a particular serum with respect to a particular Ab class that is measured. The aim of the measurement itself is then the estimation of deviation of Ag density in the microspot and the relative Ab concentration of serum Abs from the standard state. If the measurement data are suitable to identify the standard state, we can model the behavior of the system under different conditions.

The standard state also bears biological relevance. On one hand it is where the immune system is tuned to sensitively respond to changes in [Ag] in this range. On the other hand, it is this [Ab] that efficiently maintains [Ag] at the set level. Changing [Ag] will trigger B-cell receptor signals in memory B cells, leading to expansion and Ab production (47). Thus, the standard state corresponds to maximal relative thermodynamic activity and represents a steady state the immune system ideally maintains towards this particular Ag. This state is a function of B-cell differentiation, which determines affinity (50), Ag abundance, which drives B-cell proliferation (51), and immune complex removal through Fc receptors, which maintains the flow of Ag towards molecular degradation (52).

Serological measurements play a key role during pandemics in several aspects: they allow seroepidemiological monitoring of disease spread, assessment of individual responses against pathogens or vaccines and as a correlate of protection from disease. Multitudes of different assays had been developed recently for SARS-CoV-2 serology, with different platforms, Ags, methods and aims. Neutralization assays are considered good correlates of protection and they show correlations with serological measurements. The determination of Ab affinity has also been suggested to provide useful clinical information (16, 53). Unfortunately, most of these assays generate distinct results in terms of units and comparability even after transformations. An assay with truly quantitative readout of chemical properties of SARS-CoV-2 specific Abs could substantially improve our immunological understanding of COVID serology. Our observations on this limited set of serum samples already provide interesting insights. The commercial, conventional technology used here for comparison is the ELISA. The positive correlation between standard complex concentration [AbAg]° and ELISA units (**Figures 3B, C**) confirms that in spite of different detection methods the generation of bound Ab is similar. ELISA tests are adjusted with respect to coating Ag concentration and secondary reagent so as to characterize serum Ab reactivity in the relevant concentration range, which is around the standard state of our definition. Unexpectedly, the affinity and relative concentration were lower in the COVID-19 positive group in spite of increased [AbAg]° values (**Figures 3**, **4**). This result suggests that the Abs produced during the first weeks of infection are targeted against a wide range of RBD epitopes but bind with poor affinity. This is supported by other studies showing a negative impact of pre-existing common cold coronavirus immunity on SARS-CoV-2 Ab response (54–56). Antigenic sin could therefore be the reason for an increase of bound Ab molecules along with decreased average affinity and chemical potential.

The current limitation of the method with respect to analytical sensitivity is the requirement of a sufficient number of datapoints from the measurement for curve fitting. For weakly reactive (seronegative) serum samples this is a difficult task as it would require a high number of very dense Ag microspots and close to undiluted serum. As long as the characterization of sera that are positive for the given Ag is the goal it may not be a serious shortcoming but it will be more accurate to work with a method that yields quantitative results even for negative samples. With respect to reproducibility, the accuracy of the Ag density in the microspots is critical. This property is determined and can be controlled during the production of microspot arrays, so it is not influenced by the immunoassay conditions in the laboratory. This study is not a systematic assessment of SARS-CoV-2 specific humoral immune response. The results only show that SARS-CoV-2 specific immune responses can be subjected to a deep thermodynamic analysis using this technology if responses exceed a certain minimal threshold. Here we aimed to demonstrate the concept of application of physical chemistry to quantitative serology via the use of dual-titration and a normalized, generalized logistic function. Further studies using this approach will be needed to reveal the potency of the technology in discovering immunological phenomena not addressed by conventional technologies. We expect that technical improvements and larger scale production of the Ag microspot arrays will increase the efficiency of fitting and render the method suitable for routine use.

Overall, our technology is unique in the sense that it simultaneously estimates three key physico-chemical parameters relevant for immunochemical thermodynamic profiling of different Ab classes: 1) the concentration of Ag-bound Ab under standard conditions, [AbAg]°, 2) the relative concentration or thermodynamic titer [Ab]/KD, and the affinity, KD. These variables are expressed in units of molarity (M), which becomes a unitless index in the second variable, and are therefore universally comparable as long as molar concentrations of the Ag are available. With the current transition towards molecular diagnostics this is feasible for an increasing number of immunoassays. The method is based on planar protein microarray technology, which is well-established and available by now in central laboratories. We envisage the application of this technology when a thorough analysis of serological reactivity is needed. In clinical diagnostics it could follow screening steps in the diagnostic algorithm. Once diagnosis is established, the truly quantitative data should be useful for monitoring and adjusting therapy. Beyond medical serology, the generation of Ab binding data with universal units should contribute to the generation of databases for systems immunology in general.

## Data Availability

The original contributions presented in the study are included in the article/**Supplementary Material**. Further inquiries can be directed to the corresponding author.
