# Supplementary material for: In-depth immunochemical characterization of the serum antibody response using a dual-titration microspot assay
Source: Front Immunol. 2025 Feb 25;16:1494624. doi: 10.3389/fimmu.2025.1494624 (PMC11893856; doi:10.3389/fimmu.2025.1494624)
Supplement: Supplementary file 3 [file DataSheet1.pdf]

Supplementary data: Deep physico-chemical characterization of individual serum antibody responses against SARS-CoV-2 RBD using a dual titration microspot assay ÁK, ZH, KP, JZK, DNL, PS, LB, EU, TP, JP

Production and use of antigen microarrays

Microarray printing protocol

Printing was performed with a BioRad Calligrapher MiniArrayer, using 2 solid pins. Materials were printed on NHS- coated Nexterion H glass slides from Schott-Nexterion. Antigens and calibration immunoglobulin mix were prepared and diluted in printing buffer, containing 0,01% Tween20 + 2% glycerol + 0,5% DMSO in phosphate-buffered saline.

A 7x7 matrix was printed in each subarray in a 16-pad format. A 6x7 microspot matrix comprised 14 different dilutions of the antigen, each with 3 parallel spots. A 1x7 microspot row was used for the immunoglobulin reference for calibration.

Antigens printed:

1. **SARS-CoV2 Spike RBD 319-541 (OvodanBiotech)**, „RBDwuh”

The 14-point dilution series was made with a combination of a ½ and ⅓ diluting series. The final concentrations were as follows (μM)

{1}	{2}	{3}	{4}	{5}	{6}	{7}	{8}	{9}	{10}	{11}	{12}	{13}	{14}
16.666	8.333	5.555	4.166	2.083	1.851	1.041	0.617	0.520	0.260	0.205	0.130	0.068	0.065

Antibodies printed:

2. **IgA-IgM-IgG antibody mix (’AMG’) for calibration:**
   - **human IgA** (Jackson, 009-000-011)
   - **human IgM** (Sigma, I8260)
   - **human IgG** (Sigma, I2511)

The mix contains a final concentration of 10 μg/ml for each antibody. This was the 1st point of a 7-point ½-diluting series.

Slide layout with subarrays numbered from 1 to 16:

2	4	6	8	10	12	14	16
1	3	5	7	9	11	13	15

*

Subarray layout within slide

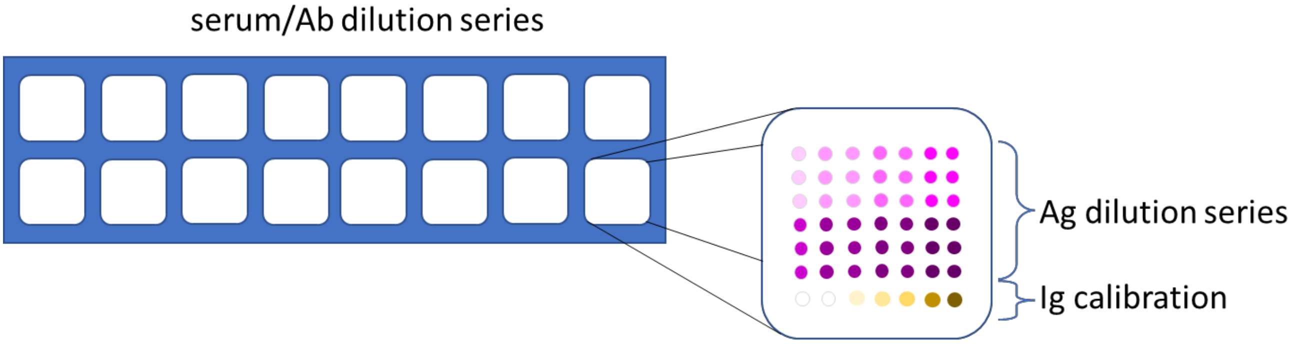

„RBD wuhan" subarray arrangement (1 subarray enlarged):

RBDwuh{14}	RBDwuh{13}	RBDwuh{12}	RBDwuh{11}		RBDwuh{10}	RBDwuh{9}	RBDwuh{8}
RBDwuh{14}	RBDwuh{13}	RBDwuh{12}	RBDwuh{11}		RBDwuh{10}	RBDwuh{9}	RBDwuh{8}
RBDwuh{14}	RBDwuh{13}	RBDwuh{12}	RBDwuh{11}		RBDwuh{10}	RBDwuh{9}	RBDwuh{8}
RBDwuh{7}	RBDwuh{6}	RBDwuh{5}	RBDwuh{4}		RBDwuh{3}	RBDwuh{2}	RBDwuh{1}
RBDwuh{7}	RBDwuh{6}	RBDwuh{5}	RBDwuh{4}		RBDwuh{3}	RBDwuh{2}	RBDwuh{1}
RBDwuh{7}	RBDwuh{6}	RBDwuh{5}	RBDwuh{4}		RBDwuh{3}	RBDwuh{2}	RBDwuh{1}
AMG{7}	AMG{6}	AMG{5}	AMG{4}		AMG{3}	AMG{2}	AMG{1}

*

Following printing slides were dried at 37°C for 60 minutes then blocked at 37°C for 60 minutes in Tris 0.1M (pH=8.0) buffer. When finished, slides were quickly rinsed in distilled water twice, then dried for 2 minutes in a fix-angled slide centrifuge. Finally, they were sealed in a light-protected bag and kept on 4°C until usage.

Microspot immunoassay protocol

Slides were taken out from 4°C and allowed to reach room temperature on the bench for 30 minutes. Slides were fitted in a Grace Biolabs steel clipped 16-chamber system for the immunoassay. Slides were rehydrated in PBS for 3x10 minutes, then blocked in PBS containing 2% bovine serum albumin for 30 minutes at 37°C. Reaction chambers were then incubated with sera of different dilutions (sample buffer 0.5% bovine serum albumin, 0.05% Tween20 in PBS), the two slides were incubated with the sera in a completely identical way.

A typical slide was assembled as follows (numbers stand for dilution factor):

spl_buffer	spl_buffer	ser_5x	ser_5x	ser_25x	ser_25x	ser_125x	ser_125x
ser_625x	ser_625x	ser_10x	ser_10x	ser_100x	ser_100x	ser_1000x	ser_1000x

Slides were washed 3x5 minutes in PBS-0.05%Tween20 on an orbital shaker.

The detection of the different Ig classes was performed with the following secondary labeled antibody mixture:

a. anti-human-**IgA** – A647 (Jackson, #:109606011, L:152435, dil:2021.02.10.) 1/1000
b. anti-human-**IgM** – Cy3 (Jackson, #:109166129, L:90910) 1/2000
c. anti-human **IgG** F(ab')$_2$ – A488 (Jackson, #:109646097, L:105228) 1/1000

Reaction layout for heavy chain detection

AMG_mix	AMG_mix	AMG_mix	AMG_mix	AMG_mix	AMG_mix	AMG_mix	AMG_mix
AMG_mix	AMG_mix	AMG_mix	AMG_mix	AMG_mix	AMG_mix	AMG_mix	AMG_mix

Incubations with the labeled antibodies diluted in sample buffer were at RT for 30 minutes.

Slides were then washed 3 x 10 min in PBS-0.05% Tween20, then completely dryed before being scanned with a Sensovation FLAIR microarray reader for the ’Blue’ channel (488nm) and a Sensovation SensoSpot microarray reader for the ’Green’ (532nm) and the ’Red’ channels (647nm).
